# Supplementary material for: Fasting mimicking diet as an adjunct to neoadjuvant chemotherapy for breast cancer in the multicentre randomized phase 2 DIRECT trial
Source: Nat Commun. 2020 Jun 23;11:3083. doi: 10.1038/s41467-020-16138-3 (PMC7311547; doi:10.1038/s41467-020-16138-3)
Supplement: Supplementary file 1 — Supplementary Information [file 41467_2020_16138_MOESM1_ESM.pdf]

Supplementary Information:

Fasting mimicking diet as an adjunct to neoadjuvant chemotherapy for breast cancer: DIRECT (BOOG 2013-04).

Stefanie de Groot et al.

Supplementary Table 1: Miller-Payne criteria for grading pathological response after neoadjuvant chemotherapy.

MP Grade	***PATHOLOGICAL CHARACTERISTICS OF THE PRIMARY TUMOR***
1	*No change or some alteration to individual malignant cells but no reduction in overall Cellularity*
2	*A minor loss of tumor cells but overall cellularity still high; up to 30% loss.*
3	*Between an estimated 30% and 90% reduction in tumor cells.*
4	*A marked disappearance of tumor cells such that only small clusters or widely dispersed individual cells remain; more than 90% loss of tumor cells*
5	*No malignant cells identifiable in sections from the site of the tumor; only vascular fibroelastotic stroma remains often containing macrophages. However, ductal carcinoma in situ may be present.*

Supplementary Table 2. Patient characteristics of both groups, and of patients who were compliant with the FMD for at least half cycles of CT or those who were not and control patients who did not fast on their own Initiative

	FMD (N=65)	***FMD-C (N=22)***	***FMD-NC (N=43)***	***Regular diet (N=64)***	***Regular diet –C (N=59)***
***Median Age (range),* Years**	49.0 (31-71)	51.0 (44-69)	49.0 (31-71)	51.0 (27-71)	51.3 (27-71)
***Median Body Mass Index (range),* kg m^{-2}**	25.7 (19.8-41.2)	25.9 (20.9-39.1)	25.4 (19.8-41.2)	26.0 (19.7-39.0)	26.3 (19.7-35.7)
WHO-status					
**Grade 0**	61 (93.8%)	20 (90.9%)	41 (95.3%)	60 (93.8%)	56 (94.9%)
**Grade 1**	3 (4.6%)	1 (4.5%)	2 (4.7%)	4 (6.3%)	3 (5.1%)
**Unknown**	1 (1.5%)	1 (4.5%)	0 (0.0%)	0 (0%)	0 (0%)
Menopausal status					
**Pre/Peri**	27 (41.5%)	11 (50.0%)	16 (37.2%)	31 (48.4%)	29 (49.2%)
**Post**	38 (58.5%)	11 (50.0%)	27 (62.8%)	31 (48.4%)	28 (47.5%)
**Unknown**	0 (0%)	0 (0%)	0 (0%)	2 (3.1%)	2 (33.9%)
T-classification					
**T1**	5 (7.7%)	1 (4.5%)	4 (9.3%)	6 (9.4%)	6 (10.2%)
**T2**	42 (64.6%)	16 (72.7%)	26 (60.5%)	41 (64.1%)	36 (61.0%)
**T3**	17 (26.2%)	4 (18.2%)	13 (30.2%)	15 (23.4%)	15 (25.4%)
**T4**	1 (1.5%)	1 (4.5%)	0 (0%)	2 (3.1%)	2 (3.4%)
N-classification					
**N0**	29 (44.6%)	8 (36.4%)	21 (48.8%)	33 (51.6%)	30 (50.8%)
**N1**	28 (43.1%)	10 (45.5%)	16 (41.9%)	26 (40.6%)	24 (40.7%)
**N2**	7 (10.8%)	3 (13.6%)	4 (9.3%)	4 (6.3%)	4 (6.8%)
**N3**	1 (1.5%)	1 (4.5%)	0 (0%)	1 (1.6%)	1 (1.7%)
Stage					
**I (ineligible)**	0 (0%)	0 (0%)	0 (0%)	1 (1.6%)	1 (1.6%)
**II**	51 (78.5%)	16 (72.7%)	35 (81.4%)	48 (75.0%)	43 (75.0%)
**III**	14 (21.5%)	6 (27.3%)	8 (18.6%)	15 (23.4%)	15 (23.4%)
HR-status					
**ER-/PR-**	14 (21.5%)	3 (13.6%)	11 (25.6%)	7 (10.9%)	6 (10.1%)
**ER-/PR unknown**	0 (0%)	0 (0%)	0 (0%)	1 (1.6%)	1 (1.7%)
**ER+/PR-**	9 (13.8%)	3 (13.6%)	6 (14.0%)	9 (14.1%)	9 (15.3%)
**ER+/PR+**	42 (64.6%)	16 (72.7%)	26 (60.5%)	47 (73.4%)	44 (74.6%)
Chemotherapy regimen					
**AC-T**	52 (80.0%)	18 (81.8%)	34 (79.1%)	47 (73.4%)	44 (74.6%)
**FEC-T**	13 (20.0%)	4 (18.2%)	9 (20.9%)	17 (26.6%)	15 (25.4%)
Grade (BR)					
**I**	2 (3.1%)	0 (0.0%)	2 (4.7%)	2 (3.1%)	2 (3.4%)
**II**	43 (66.2%)	15 (68.2%)	28 (65.1%)	42 (65.6%)	37 (62.7%)
**III**	20 (30.8%)	7 (31.8%)	13 (30.2%)	19 (29.7%)	19 (32.2%)
**Unknown**	0 (0%)	0 (0%)	0 (0%)	1 (1.6%)	1 (1.7%)
Tumortype					
**Ductal**	53 (81.5%)	18 (81.8%)	35 (81.4%)	49 (76.6%)	44 (74.6%)
**Lobular**	9 (13.8%)	4 (18.2%)	5 (11.6%)	13 (20.3%)	13 (22.0%)
**Other**	3 (4.6%)	0 (0.0%)	3 (7.0%)	2 (3.1%)	2 (3.4%)

Patient characteristics. Abbreviations: CT: chemotherapy FMD: Fasting mimicking diet, C: compliant, NC: Not compliant, HR: hormone receptor, AC-T: doxorubicin/cyclophosphamide followed by docetaxel, FEC-T: Fluorouracil/epirubicin/cyclophosphamide followed by docetaxel, BR: Bloom Richardson, ER: estrogen receptor, PR: progesterone receptor.

Supplementary Table 3. Compliance in both arms

	FMD (N=65)	***Regular diet (N=64)***
Compliant – all cycles and surgery		
**Yes**	10 (15.4%)	59 (92.2%)
**No**	55 (84.6%)	5 (7.8%)
Compliant – all cycles*		
**Yes**	13 (20.0%)	
**No**	52 (80.0%)	
Compliant – half of cycles**		
**Yes**	22 (33.8%)	
**No**	43 (66.2%)	
Compliant – first cycle		
Yes	53 (81.5%)	
No	11 (16.9%)	
Unknown	1 (1.5%)	
Reason for early stop FMD		
**Taste**	26 (51.0%)	
**Nausea**	10 (15.4%)	
**Hunger**	5 (9.8%)	
**Stop chemotherapy**	2 (3.9%)	
**Other**	8 (35.3%)	

*Compliance given per group. * Compliance is defined as patients complied the FMD or regular diet all cycles of their treatment arm. ** Compliance is defined as patients complied to the FMD or regular diet half of the cycles of their treatment arm. Regular diet patients were not compliant if they were fasting for at least one cycle on their own. Abbreviations: FMD: Fasting mimicking diet.*

Supplementary Table 4. Grade III/IV toxicity during 8 cycles of AC-T/FEC-T in both groups

Grade III/IV	***FMD (N=65)***	***Control (N=64)***	***P- value (ITT)***
Total	*49 (75.4%)*	*42 (65.6%)*	*0.224*
Neutropenic fever	*22 (33.8%)*	*12 (18.8%)*	*0.052*
Neutropenia	*33 (50.8%)*	*22 (34.4%)*	*0.060*

Grade III/IV side effects were scored according CTCAE4.03. Each side effect was scored maximal once per patient during the course. FMD; fasting mimicking diet, C: compliance, ITT: intention to treat. Crosstabs were used.

Supplementary Table 5. Associations between tumor, patient characteristics, treatment arm and efficacy of chemotherapy

Parameter		***N***	***pCR***	***Univariate analysis***	***P value***	***Multivariate analysis***	***P value***
BMI	*< 25kg m-2*	59	11 (18.6%)	1.000	***0.032***	1.000	*0.067**
	≥ 25kg m-2	69	4 (5.8%)	0.269 (0.081-0.895)		0.308 (0.087-1.085)	
HR receptor status	*ER +*	106	8 (7.5%)	1.000	***0.003***	1.000	***0.006****
	ER -	22	7 (31.8%)	5.717 (1.809-18.07)		5.626 (1.624-19.48)	
Stage	*I/II*	100	13 (13.0%)	1.000	*0.402*		
	III	28	2 (7.1%)	0.515 (0.109-2.430)			
CT schedule	*AC-T*	98	14 (14.3%)	1.000	*0.136*		
	FEC-T	30	1 (3.3%)	0.207 (0.026-1.643)			
ITT	*FMD*	65	7 (10.8%)	0.830 (0.282-2.442)	*0.735*	0.581 (0.174-1.942)	*0.378*
	Regular diet	63	8 (12.7%)	1.000		1.000	
PP	*FMD C*	22	3 (13.6%)	1.150 (0.269-4.911)	*0.850*	1.074 (0.216-5.344)	*0.816*
	FMD NC	43	4 (9.3%)	0.747 (0.204-2.734)	*0.660*	0.478 (0.113-2.017)	*0.315*
	Regular diet C	58	7 (12.1%)	1.000		1.000	
Parameter		***N***	***MP 4/5 vs. 1/2/3***	***Univariate analysis***	***P value***	***Multivariate analysis***	***P value***
BMI	*< 25kg m-2*	59	22 (37.3%)	1.000	***0.013***	1.000	***0.016****
	≥ 25kg m-2	69	12 (17.4%)	0.354 (0.157-1.801)		0.354 (0.152-0.825)	
HR receptor status	*ER +*	106	24 (22.6%)	1.000	***0.032***	1.000	*0.082**
	ER -	22	10 (45.5%)	2.847 (1.096-7.396)		2.437 (0.894-6.646)	
Stage	*I/II*	100	30 (30.0%)	1.000	*0.105*		
	III	28	4 (14.3%)	0.389 (0.124-1.218)			
CT schedule	*AC-T*	98	26 (26.5%)	1.000	*0.988*		
	FEC-T	30	8 (26.7%)	1.007 (0.399-2.540)			
ITT	*FMD*	65	20 (30.8%)	1.556 (0.703-3.441)	*0.275*	1.526 (0.655-3.556)	*0.328*
	Regular diet	63	14 (22.2%)	1.000		1.000	
PP	*FMD C*	22	10 (45.5%)	3.194 (1.115-9.152)	***0.031***	4.109 (1.297-13.02)	***0.016***
	FMD NC	43	10 (23.3%)	1.162 (0.449-3.006)	*0.757*	0.926 (0.328-2.614)	*0.884*
	Regular diet C	58	12 (20.7%)	1.000		1.000	
Parameter		***N***	***Radiological response CR+PR vs. SD+PD***	***Univariate analysis***	***P value***	***Multivariate analysis***	***P value***
BMI	*< 25kg m-2*	50	43 (86.0%)	1.000	*0.214*		
	≥ 25kg m-2	55	42 (76.4%)	0.526 (0.191-1.448)			
Stage	*I/II*	83	67 (80.7%)	1.000	*0.907*		
	III	22	18 (81.8%)	1.075 (0.320-3.614)			
HR receptor status	*ER +*	86	71 (82.6%)	1.000	*0.376*		
	ER -	19	14 (73.7%)	0.592 (0.185-1.893)			
CT schedule	*AC-T*	82	68 (82.9%)	1.000	*0.334*		
	FEC-T	23	17 (73.9%)	0.583 (0.195-1.742)			
ITT	*FMD*	53	47 (88.7%)	2.886 (1.012-8.227)	***0.047***	3.168 (1.062-9.446)	***0.039***
	Regular diet	52	38 (73.1%)	1.000		1.000	
PP	*FMD C*	19	18 (94.7%)	6.882 (0.832-56.91)	***0.074***	6.365 (0.759-53.37)	*0.088*
	FMD NC	34	29 (85.3%)	2.218 (0.706-6.963)	*0.172*	2.715 (0.785-9.390)	*0.115*
	Regular diet C	47	34 (72.3%)	1.000		1.000	

*Regression models are used to measure associations. The radiological response was scored according RECIST and given for complete response + partial response vs. stable disease +progression disease. Abbreviations: pCR: pathological complete response, BMI: body mass index, HR: hormone receptor status, FMD: fasting mimicking diet, C: Compliant, NC: Not compliant, ITT: Intention to treat, PP: Per protocol, MP: Miller and Payne, CR: complete response, PR: partial response, SD: stable disease, PD: progression disease. * Result from ITT multivariate analysis.*

Supplementary Table 6. Metabolic and endocrine parameters first cycle

Parameter	***FMD (N=65)***	***FMD-C (N=53)***	***FMD-NC (N=11)***	***Regular diet (N=64)***	***Regular diet-C (N=59)***	***P- value (ITT)***	***P- value (PP)***
Glucose (3.1-6.4mmol/L)	5.1	*4.9*	*6.4*	5.7	*5.9*	***0.062***	***0.006***
Insulin (0-20mU/L)	6.0	*4.0*	*19.0*	12.0	*13.5*	***0.004***	***<0.0001***
IGF-1 (5.4-24.3nmol/L)	17.4	*17.3*	*20.0*	17.9	*18.0*	*0.925*	*0.602*
IGF-BP3 (2.2-5.8mg/L)	3.7	*3.7*	*3.8*	4.0	*4.0*	*0.601*	*0.570*
CRP (<5.0mg/L)	3.0	*3.5*	*3.0*	2.0	*2.0*	***0.007***	***0.010***
Ketone bodies							
Pos	32 (74.4%)	*14 (93.3%)*	*10 (35.7%)*	5 (12.5%)		***<0.0001***	***<0.0001***
Neg	11 (25.6%)	*1 (6.7%)*	*18 (64.3%)*	35 (87.5%)			

Insulin-like growth factor 1, IGF-BP3 insulin- like growth factor binding protein 3. ITT: intention to treat, PP: per protocol. Independent t-tests and Mann-Whitney tests were used

Supplementary Table 7. γ-H2AX intensity in peripheral blood mononuclear cells

Parameter	***N***	***Before CT (SE)***	***3 hours after CT (SE)***	***P value each group***	***P value between groups***
CD45+CD3+ T-lymphocytes	*FMD (n = 16)*	143.4 (10.0)	173.4 (16.4)	***0.011***	***0.045***
	Regular diet (n = 11)	147.7 (8.6)	206.8 (14.6)	***0.001***	
CD45+CD14+CD15- monocytes	*FMD (n = 16)*	229.1 (16.0)	273.2 (21.5)	***0.025***	*0.388*
	Regular diet (n = 10)	230.7 (11.1)	309.5 (30.9)	***0.021***	
CD45+CD3- non-T cells	*FMD (n = 16)*	175.7 (10.6)	231.0 (21.3)	***0.025***	*0.856*
	Regular diet (n = 11)	185.6 (9.4)	237.8 (23.9)	***0.037***	

Paired comparison between pre- and 3 hours post- chemotherapy for different cell types in peripheral blood mononuclear cells. γ-H2AX intensity is given as mean. FMD; fasting mimicking diet. Independent and paired t-tests were used.

Supplementary figure 1.

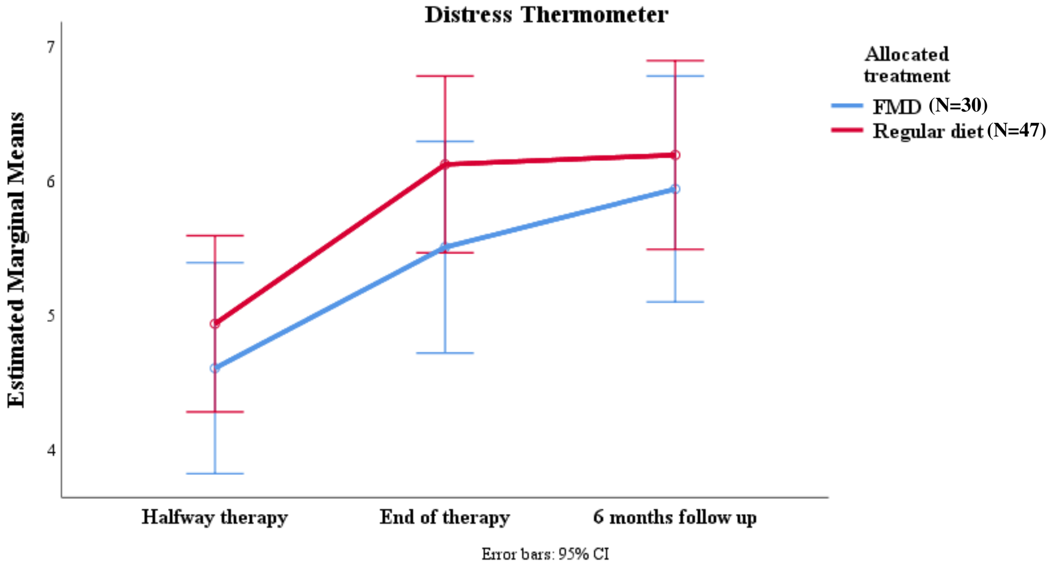

Psychosocial distress given for 3 timepoints: halfway therapy, at the end of therapy and at six months follow-up. Error bars indicate the 95%CI of the mean. FMD: fasting mimicking diet. CI: confidence interval. FMD N=30 and regular diet N=47. Source data are provided as a Source Data file.

Supplementary figure 2.

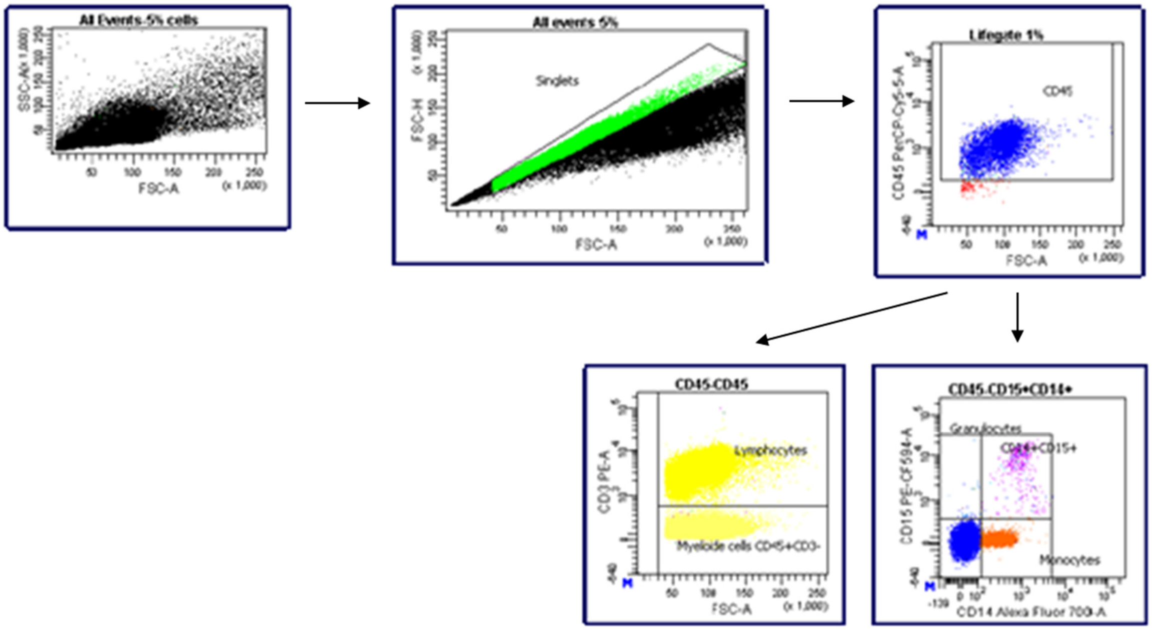

Gating strategy: The CD45+ cells were gated, after which the CD3+ T-lymphocytes, CD3- non-T cells (also harboring B lymphocytes) or CD14+CD15- monocytes were analyzed for the geomean (as measure for the intensity) of γ-H2AX.

Supplementary figure 3.

Chemolieve™	Day 1	Day 2		Day 3		Day 4 *	Day 5	Day 6
Morning	Tea	Energy Drink	Tea	Energy Drink	Tea	Tea	NR-1 (2)**	Bar
	Bar						TRANSITION DIET	Algal Oil (1)
	Algal Oil (2)							NORMAL DIET
Lunch	Soup		Soup		Soup	Soup		
	Chips							
	NR-1(2)							
Afternoon	Tea		Tea		Tea	Tea		
	Bar							
Dinner	Soup		Broth		Broth	Broth		
	Chips							

Chemolieve fasting mimicking diet schedule.
