# Supplementary material for: Fasting mimicking diet as an adjunct to neoadjuvant chemotherapy for breast cancer in the multicentre randomized phase 2 DIRECT trial
Source: Nat Commun. 2020 Jun 23;11:3083. doi: 10.1038/s41467-020-16138-3 (PMC7311547; doi:10.1038/s41467-020-16138-3)
Supplement: Supplementary file 3 — Reporting Summary [file 41467_2020_16138_MOESM3_ESM.pdf]

nature research

Corresponding author(s): J.R. Kroep

Last updated by author(s): Feb 10, 2020

Reporting Summary

Nature Research wishes to improve the reproducibility of the work that we publish. This form provides structure for consistency and transparency in reporting. For further information on Nature Research policies, seeAuthors & Referees and theEditorial Policy Checklist .

Statistics

For all statistical analyses, confirm that the following items are present in the figure legend, table legend, main text, or Methods section.

n/a	Confirmed	
☐	☒	The exact sample size (*n*) for each experimental group/condition, given as a discrete number and unit of measurement
☐	☒	A statement on whether measurements were taken from distinct samples or whether the same sample was measured repeatedly
☐	☒	The statistical test(s) used AND whether they are one- or two-sided<br>*Only common tests should be described solely by name; describe more complex techniques in the Methods section.*
☐	☒	A description of all covariates tested
☐	☒	A description of any assumptions or corrections, such as tests of normality and adjustment for multiple comparisons
☐	☒	A full description of the statistical parameters including central tendency (e.g. means) or other basic estimates (e.g. regression coefficient) AND variation (e.g. standard deviation) or associated estimates of uncertainty (e.g. confidence intervals)
☐	☒	For null hypothesis testing, the test statistic (e.g. *F*, *t*, *r*) with confidence intervals, effect sizes, degrees of freedom and *P* value noted<br>*Give P values as exact values whenever suitable.*
☒	☐	For Bayesian analysis, information on the choice of priors and Markov chain Monte Carlo settings
☒	☐	For hierarchical and complex designs, identification of the appropriate level for tests and full reporting of outcomes
☐	☒	Estimates of effect sizes (e.g. Cohen's *d*, Pearson's *r*), indicating how they were calculated

Our web collection on statistics for biologists contains articles on many of the points above.

Software and code

Policy information about availability of computer code

Data collection	The web based relational database management system ProMISe was used for data storage and exchange.
Data analysis	All data were analyzed using IBM SPSS Statistics for Windows (Version 23.0. Armonk, NY: IBM Corp). Also data were analyzed using BD FACS Diva Software version 6.2

For manuscripts utilizing custom algorithms or software that are central to the research but not yet described in published literature, software must be made available to editors/reviewers. We strongly encourage code deposition in a community repository (e.g. GitHub). See the Nature Research guidelines for submitting code & software for further information.

Data

Policy information about availability of data

All manuscripts must include a data availability statement. This statement should provide the following information, where applicable:

- Accession codes, unique identifiers, or web links for publicly available datasets
- A list of figures that have associated raw data
- A description of any restrictions on data availability

All study data are presented in the manuscript and supplementary materials. The source data underlying Table 1-2, Figure 2-6, Supplementary figure 1 and Supplementary table 2-7 are provided as a Source Data file. Additional raw data that support the findings of this study are available from the corresponding author upon reasonable request

Field-specific reporting

Please select the one below that is the best fit for your research. If you are not sure, read the appropriate sections before making your selection.

Life sciences study design

All studies must disclose on these points even when the disclosure is negative.

Sample size	The primary endpoint of phase II of the study was grade III/IV toxicity. Based on trials with similar neo-adjuvant chemotherapy, the statistical power analysis revealed that a total number of 128 patients (64 patients in each arm) was required to be able to detect a 50% reduction of grade III/IV adverse effects with 80% power using a nominal significance level of 3.06% .<br>The primary endpoint of the phase III part of the study was pathological complete response (pCR). We estimated the overall pCR rate to amount to 18%, based on studies examining similar third generation chemotherapy.
Data exclusions	No data were excluded from the analysis
Replication	The findings of this trial are not replicated or reproduced because there was not enough material.
Randomization	Patients were centrally randomized at the LUMC datacenter through block randomization with various block sizes stratified by stage (II versus III), estrogen receptor status (positive versus negative), BMI (<25kg/m2 versus >25kg/m2) and chemotherapy regimen (AC-T versus FEC-T).
Blinding	Patients and their physicians could not be blinded because the experimental arm with fasting cannot be controlled with a placebo.<br>Data collection and analysis were performed blinded for the allocated study arm

Reporting for specific materials, systems and methods

We require information from authors about some types of materials, experimental systems and methods used in many studies. Here, indicate whether each material, system or method listed is relevant to your study. If you are not sure if a list item applies to your research, read the appropriate section before selecting a response.

Materials & experimental systems

n/a	Involved in the study	
☒	☐	Antibodies
☒	☐	Eukaryotic cell lines
☒	☐	Palaeontology
☒	☐	Animals and other organisms
☐	☒	Human research participants
☐	☒	Clinical data

Methods

n/a	Involved in the study	
☒	☐	ChIP-seq
☐	☒	Flow cytometry
☒	☐	MRI-based neuroimaging

Human research participants

Policy information about studies involving human research participants

Population characteristics	Dutch patients with HER2-negative, stage II/III early breast cancer, age ≥ 18 years, BMI > 19kg/m2, WHO performance state 0-2, absence of diabetes mellitus with normal bone marrow reserve, adequate liver and renal function and normal cardiac function
Recruitment	Patients were recruited during their first visit with their oncologist within 4 weeks before the start of chemotherapy
Ethics oversight	The study (NCT02126449) was conducted in accordance with the Declaration of Helsinki (October 2013) and approved by the Ethics Committee of the Leiden University Medical Center in agreement with the Dutch law for medical research involving human subjects.

Note that full information on the approval of the study protocol must also be provided in the manuscript.

Clinical data

Policy information about clinical studies

All manuscripts should comply with the ICMJEguidelines for publication of clinical research and a completedCONSORT checklist must be included with all submissions.

Clinical trial registration	NCT02126449
Study protocol	The full trial protocol has been shared with Nature communications, and is added as supplemetary file.
Data collection	Prior to study entry (at least 4 days before start chemotherapy), patients will undergo a standard workup including the following assessments:<br>• Complete history and physical examination including, but not limited to, vital<br>signs, height, weight, BMI, WHO performance status and any observational tumor measurements<br>• Hematology: Hemoglobin, WBC and differential, platelet count

• Biochemistry: Na, K, phosphate, Mg, Ca, vitamin D, albumin, serum creatinine, ASAT, ALAT, alkaline phosphatase, bilirubin, LDH.
• Bilateral mammography and ultrasound of the axilla and breast with fine needle aspiration of the primary tumor and in case of any suspected lymph node metastases
• Electrocardiogram
• MRI for tumor measurements
Placement of a marker in the originally tumor bed to guide pathology examination after surgery, to facilitate sparing surgery in case of a complete remission and to facilitate radiotherapy planning.)
• Chest X-ray, ultrasound of the liver and bone scan or PET/CT scan to exclude distant metastases according to Dutch National Guidelines

Pathologic measurements:

Primary core biopsy:
One initial core biopsy will be taken, using a 22mm 14-Gauche needle. The material should be fixed in 4% buffered formalin and processed according to standard procedures. Standard H&E, and immunohistochemistry for ER and PR will performed. HER2 will be assessed according to the national consensus.
Operation specimen:
The primary tumor needs to be marked before starting chemotherapy to accurate localize the original tumor bed in order to allow definite pathologic analyses. The clips need to be visible during surgery in order to facilitate sparing surgery in case of a complete remission. The resection specimen will be processed and evaluated in a standardized fashion. The specimen is received unfixed. After inking of the resection margin a piece of suspected tumor tissue is snap frozen. Next the specimen is sliced at 0.5 cm, and fixed for at least 24 hours. Gross pictures will be taken. The tissue will be evaluated macroscopically and one block per centimeter of tumor will be taken; if no tumor is recognized at least 5 blocks tissue should be taken. One H&E section is taken per block, and when no tumor cells are found immunohistochemistry will be performed on those blocks in which tumor regression is recognized. Tumor regression scored along published guidelines including the Miller and Payne system. Sentinel nodes and other axillary nodes will be processed according to the national guideline.

Clinical response measured by MRI
MRI imaging of the effected breast will be scheduled immediately prior to the first cycle of chemotherapy (cycle 1) and halfway the neoadjuvant treatment (after the last AC or FEC cycle). The MRI tomographic findings will be evaluated by an expert panel. Based on the breast MRI findings, patients will be classified (RECIST) according to the degree of documented size reduction into groups with progressive disease (PD), no response (NR), partial response (PR) or complete response (CR).

Blood sampling
Before Inclusion:
Kidney and liver function, counts of white blood cells (WBCs) and differentiation, platelets, RBCs, hemoglobin and hematocrit will be performed in all subjects before inclusion. Also in this sample, FSH and estradiol will be performed to determine the menopausal status.
Before every cycle (routine):
Electrolytes, kidney and liver function, counts of white blood cells (WBCs) and differentiation, platelets, RBCs, hemoglobin and hematocrit will be performed in all subjects before chemotherapy is administered on day 0 during a routine vena puncture (normal procedure). Also in this sample, fasting values of glucose, insulin, IGF-I, and CRP will be performed to determine the body's response to fasting (for control group, non-fasting values).

Before cycle 1, before the last AC or FEC cycle and before the last docetaxel cycle (routine):
IGF-BP3, FT4, T3, TSH will be performed to determine the body's response to fasting during cycles 1, 4 and 8(for control group, non-fasting values) during a routine vena puncture (normal procedure).
Before cycle 1 (SNPs):
Before cycle one during a routine vena puncture planned for other medical reasons 1 extra blood samples (5ml EDTA) will be taken to identify SNPs that can be used as biomarkers to predict treatment outcome.
Before and 3 hours after start of cycles 1 and before the last AC or FEC cycle (DNA damage and nutrient sensing optional in LUMC):
At chemotherapy cycle 1 and before the last AC or FEC cycle, optional 4 extra blood samples (10 ml heparin tubes) will be taken to determine chemotherapy-induced DNA damage and nutrient sensing systems in leukocytes before (baseline value) and 3 hours after start of chemotherapy infusion

Quality of life

To measure Quality of Life (QoL) and illness perceptions patients will be asked to fill out questionnaires about their well-being and their experienced side effects of chemotherapy
Questionnaire 1
Consists of EORTC QLQ-C30, EORTC BR23 and the B-IPQ will be filled out before the start of chemotherapy but after randomization. The questionnaire will be given to the patient in the local hospital after randomization.

Questionnaire 2
Consists of EORTC QLQ-C30, EORTC BR23 and distress thermometer and will be filled out before the last AC or FEC cycle . The

	questionnaire will be sent to the patient from the LUMC datacenter.<br><br>Questionnaire 3<br>Consists of EORTC QLQ-C30, EORTC BR23, distress thermometer and B-IPQ and will be filled out before the last docetaxel cycle . The questionnaire will be sent to the patient from the LUMC datacenter.<br><br>Questionnaire 4<br>Consists of EORTC QLQ-C30, EORTC BR23 and distress thermometer and will be filled out six months after surgery. The questionnaire will be sent to the patient from the LUMC datacenter.
Outcomes	The primary endpoint of the phase II and phase III parts of the study were grade III/IV toxicity and pathological complete response (pCR), respectively. Toxicity was documented by the physician and graded according to the Common Terminology Criteria for Adverse Events version4.03 (CTCAE v.4.03). Pathological complete response (pCR) was defined as the absence of residual invasive cancer within the breast and lymph nodes , excluding isolated tumor cells (ITC).<br>Secondary endpoints included radiological response and pathological response according to the Miller and Payne. Histopathology was centrally revised by one pathologist (DC), who was blinded to which treatment the patient received. Clinical response was measured by MRI or ultrasound of the breast halfway and at the end of therapy, according to RECIST1.120.

Flow Cytometry

Plots

Confirm that:

- [x] The axis labels state the marker and fluorochrome used (e.g. CD4-FITC).
- [x] The axis scales are clearly visible. Include numbers along axes only for bottom left plot of group (a 'group' is an analysis of identical markers).
- [x] All plots are contour plots with outliers or pseudocolor plots.
- [x] A numerical value for number of cells or percentage (with statistics) is provided.

Methodology

Sample preparation	PBMCs were isolated as decribed before11
Instrument	BD LSR Fortessa Flow Cytometer analyzer, BD Bioscience, Breda, The Netherlands
Software	BD FACS Diva Software version 6.2
Cell population abundance	We measured more than 1.000.000 cells.
Gating strategy	The CD45+ cells were gated, after which the CD3+ T-lymphocytes, CD3- myeloid cells (also harboring B lymphocytes) or CD14 +CD15- monocytes were analyzed for the geomean (as measure for the intensity) of γ-H2AX

- [x] Tick this box to confirm that a figure exemplifying the gating strategy is provided in the Supplementary Information.
